# Effects of a food enriched with probiotics on *Streptococcus mutans* and *Lactobacillus* spp. salivary counts in preschool children: a cluster randomized trial

**DOI:** 10.1590/1678-7757-2017-0318

**Published:** 2018-05-03

**Authors:** Judy Villavicencio, Lina Maria Villegas, Maria Cristina Arango, Susana Arias, Francia Triana

**Affiliations:** 1 Escuela de Odontología, Universidad del Valle, Cali, Colombia; 2 Escuela de Ciencias Básicas, Universidad del Valle, Cali, Colombia

**Keywords:** Preschool children, Probiotics, Lactobacillus rhamnosus, Bifidobacterium longum, Streptococcus mutans, *Lactobacillus* spp, Caries prevention, Early childhood caries, Clinical trial

## Abstract

Probiotics have provided benefits to general health, but they are still insufficient to dental health.

**Objective:**

This study aimed to evaluate milk supplemented with probiotic bacteria and standard milk, measured by levels of *Streptococcus mutans* (*S. mutans*) and *Lactobacillus* spp., in 3-4-year-old children after 9 months of intervention.

**Material and Methods:**

The study was a triple-blind, placebo-controlled, randomized trial. The sample was composed of 363 preschoolers attending five child development centers in Cali, Colombia. They were randomized to two groups: children in the intervention group drank 200 mL of milk with *Lactobacillus rhamnosus* 5x10^6^ and *Bifidobacteruim longum* 3x10^6^, and children in the control group drank 200 mL of standard milk. Interventions occurred on weekdays and information was gathered through scheduled clinical examination. The primary result was the number of colony forming units (CFU) of *S. mutans* and *Lactobacillus* spp. in the saliva. Secondary results were dental caries, rated by the International Caries Detection and Assessment System (ICDAS), dental plaque, pH, and salivary buffer capacity.

**Results:**

The proportion of *S. mutans* was lower in the intervention group compared with the control group after 9 months; however, the differences did not reach statistical significance (p=0.173); on the other hand, statistically significant differences between groups were found in the CFU/mL of *Lactobacillus* spp. (p=0.002). There was not statistically significant difference in the prevalence of dental caries for both groups (p=0.767). Differences between groups were found in the salivary buffering capacity (p=0.000); neither salivary pH nor dental plaque were significantly different.

**Conclusions:**

Regular consumption of milk containing probiotics bacteria reduced CFU/mL of *Lactobacillus* spp. and increased salivary buffering capacity at 9 months of consumption.

## Introduction

Early childhood caries (ECC) is one of the most prevalent and costly health conditions among children^26^. Regional data of the IV National Oral Health Survey conducted in Colombia in 2014 (ENSAB IV)^7^ reported a prevalence of 29.31% in 1-year-old children, an increased prevalence of 83.03% in 3-year-olds and 88.83% in 5-year-olds. These results show the importance of investigating new self-administrated preventive measures that could be added to existing evidence-based recommendations to control ECC^6^.

Probiotics are live microorganisms that confer health benefits to the host when administered in adequate amounts^28^. There are reports on gastrointestinal and urogenital problems, allergic diseases, and more generally to enhance the function of the digestive tract and promote the immune system^8^. The mechanism of probiotics in the oral cavity is not completely understood, but they are associated with reductions in CFU counts of cariogenic pathogens^20^. Additionally, probiotics modulate the inflammatory responses, both humoral and cellular, and products as lactic acid, hydrogen peroxide, and bacteriocins, which are antimicrobial agents produced by lactic acid bacteria^20^ ^,^ ^21^ ^,^ ^30^. Most of the studies reviewed mention the ability of the lactic acid to compete with pathogens for adhesion surfaces and nutrients, causing the displacement of the latter^20^.

The therapeutic potential of probiotics against tooth decay has been studied. This concept is based on the idea of maintaining or restoring the natural oral microbiome by interfering and/or inhibiting pathogenic bacteria^4^. The use of probiotics to manage the oral microflora appears to be an effective method to control oral conditions^20^.

Clinical studies have revealed that strains such as *Lactobacillus rhamnosus* GG, *Lactobacillus reuteri, Lactobacillus casei, Lactobacillus paracasei, Lactobacillus acidophilus La-5, Lactobacillus brevis CD2, Bifidobacterium animalis* subsp*. lactis* BB-12, *Bifidobacterium longum*, *Bifidobacterium bifidum, Bifidobacterium lactis, Bifidobacterium infantis,* and *Saccaromyces cereviasae* are associated with decreases in the number of *S. mutans* counts in dental plaque and saliva^11^ ^,^ ^17^ ^,^ ^22^ ^,^ ^24^ ^,^ ^25^. Regarding the *Lactobacillus* spp., the results are less decisive^4^ ^,^ ^22^ ^,^ ^25^.

Clinical trials investigated the effect of probiotics on caries prevalence as a final goal in preschool children^9^ ^,^ ^10^ ^,^ ^17^ ^,^ ^22^ ^–^ ^24^. Six of these studies reported a reduction in caries occurrence after probiotic exposure compared with control groups in children^10^ ^,^ ^17^ ^,^ ^19^ ^,^ ^22^ ^–^ ^24^; Hasslöf, et al.^9^ (2013) did not find statistically significant differences in caries diagnoses and *S. mutans* or *Lactobacillus* spp. counts in a long term. There is no information available in Colombia about the effect of the administration of daily probiotics in a population of preschoolers with high caries levels and the presence of *S. mutans* and *Lactobacillus* spp. counts as related to the occurrence of ECC. The purpose of this study was to evaluate milk supplemented with probiotic bacteria and standard milk, measured by levels of *S. mutans* and *Lactobacillus* spp., in 3-4-year-old children after 9 months of intervention. While the non-existence of variations between milk supplemented with probiotic bacteria and standard milk by measuring *S. mutans* and *Lactobacillus* spp. counts in 3-4-year-old children, the hypothesis was null.

## Material and methods

### Subjects

Three- to four-year-old children participated in this trial. Participants were recruited from five Child Development Centers in a hillside area of Cali, Colombia. They all had parental permission and consent to participate in the study. The children underwent supervised tooth brushing with fluoride toothpaste (1450 ppm) at least once a day, at the day care centers. Healthy children, without systemic disorders, neither milk intolerance nor under permanent antibiotic treatment reported by their parents, were included in this study.

### Study design, sample size, randomization, and blinding

The study was planned as a cluster randomized, triple-blind, placebo-controlled, two-arm trial and interventions were carried out between September 2016 and June 2017. The published protocol refers to a 12-month period, however, considering the Colombian day care centers, we had to modify the original 12-month period to a 9-month alternative period (ANZCTR-ACTRN12616001363471, http://www.anzctr.org.au).

Using a random number generator, one of the authors (J. V.) assigned each group of the Child Development Centers either to the intervention or control group. They were allocated to a blue or black code to hide their identity. The code was only known by the lead author (J. V.) and was not revealed until all data were analyzed. Neither the researchers, clinicians, the staff of the Child Development Centers nor the relatives of the participants knew whether the children were receiving the probiotic-supplemented milk or the control. The sample type was probabilistic by clusters. The sampling frame corresponds to the groups of the five Child Development Centers, conformed by 10, which will be the primary sampling units. At random, the ten groups were selected for each site (secondary sampling unit). The calculation of the sample was done with Epidat version 3.4, based on the 1,871 children served in the development centers in the hillside of Cali. They were tested with a confidence level of 90%, p-value of 0.80, alpha level of 10%, and expected frequency of 50% for a total of 328 children. The estimated proportion of attrition rate was determined in 10% *per* year. Therefore, 10% of oversampling was performed to compensate for this of 33 children.

This study was carried out in accordance with the Declaration of Helsink of 1964. Parents of all children signed the informed consent form prior to inclusion in the study. The Institutional Review Committee of Human Ethics of the Universidad del Valle, Faculty of Health, approved the protocol of this project through Act number. 009-015.

### Intervention

Children were given 200 mL of milk during breakfast every day, for five days a week, during a 9-month period. The probiotic-supplemented milk and the placebo milk were prepared and given to the children only on weekdays. The nursery school staff prepared the milk with the researcher's supervision. For the probiotic group, 210 mL of water at 40°C that had been previously boiled was added to seven tablespoons of powdered milk (Nestlé Nan Pro 3®, Nestlé Mexico S.A). For the placebo group, 180mL of water at 40°C that had been previously boiled was added to six tablespoons of powdered milk (Alpina Baby+Plus 3®, made in Switzerland). The difference in the preparation was because the probiotic milk and the placebo milk were of different brands. According to the manufacturer of probiotic milk, the concentration of *Lactobacillus rhamnosus* was 5x10^6^ CFU/g and of the *Bifidobacterim longum* 3x10^6^ CFU/g.

### Check of Compliance

Consumption of milk and absence for sickness or any other situations of the children were daily monitored.

### Clinical Examination and Data Collection

Clinical dental evaluation was carried out at baseline and during the 9-month period of study. One dentist using an artificial light, a mouth mirror, and a rounded tip explorer examined all children in the child development centers. ICDAS criteria were used for detecting visual and tactile dental lesions and for describing their severity^18^. An expert clinician certified by the ICDAS Committee performed the exams in the children (Kappa intra-examiner 0.85 and inter-examiner 0.72). ICDAS codes 2 to 6 were used to measure caries increments. Category one lesions were excluded because of their difficult diagnosis at these stages. If a tooth presented two ICDAS diagnoses, the one with the highest degree of severity was recorded^18^ ^.^ The bacterial plaque was recorded according to the modified Silness-Löe index, with a surface free of plaque calibrated to the value 1, for plaque visible only at the passage of the instrument, and 2 for plaque visible to the naked eye^15^.

### Collection and culturing of salivary samples

A pediatric dentist was responsible for taking the sample of unstimulated saliva to each child. The deposit was made between one and two milliliters of saliva using a 50 ml conical sterile plastic tube, marked with a study entry code for each child. The media selected for isolation of *S. mutans* were Agar Mitis salivarius with tellurium and bacitracin (MSTB) and MRS agar (Man, Rogosa and Sharpe) for *Lactobacillus* spp^14^. The 0.1 ml saliva sample was diluted with 0.9 ml of sterile normal saline; next, decimal dilutions of saliva were made up to 10^-3^. Each medium plate was cultured from 0.1 ml of the previously diluted sample to 10^-3^; then, the sample was uniformly spread. Finally, the plates were sealed. The media were incubated via anaerobically system (gas-pack jars) with a mixture of 95% N_2_ and 5% CO_2_ for 48 hours at 37°C^16^. The bacterial colonies were observed under a stereo microscope and identified by morphology and color. The CFU was multiplied by the number of times the sample was originally diluted and expressed as the number of CFU/mL of saliva. For *S. mutans* and *Lactobacillus* spp., total CFU/mL counts were taken.

The pH of the saliva was measured with a digital pH meter in the laboratory. The buffer capacity of the saliva was obtained by adding hydrochloric acid (HCl) to it, in a 1:3 relation. The final pH values obtained were evaluated in ranges to obtain levels of the saliva buffer capacity, with values <3.5 indicating very low capacity; 3.50-4.24 low capacity; 4.25-4.75 normal capacity; and higher than 4.75 high capacity^13^.

### Outcome Measures

The primary result was to measure the CFU of *S. mutans* and *Lactobacillus* spp. from saliva. Secondary results were the presence of dental caries lesions, which was assessed using ICDAS, dental plaque, pH, and salivary buffer capacity in 3-4-year-old preschool children.

### Statistical Analysis

Data were recorded with Microsoft Excel and validated through EpiData 3.1 software. The analyses were performed using the statistical package IBM SPPS^®^ Statistics 21 version. Univariate descriptive analyses were performed by noting means, standard deviations, medians, and interquartile ranges according to the normality; variables were then tabulated to obtain the groups of children supplemented with probiotic milk and those supplemented with standard milk. The dependent variables were the changes of *S. mutans* and *Lactobacillus* spp. A Mann Whitney U test for numerical variables and X^2^ test for categorical variables was performed.

## Results

**Figure 1:**
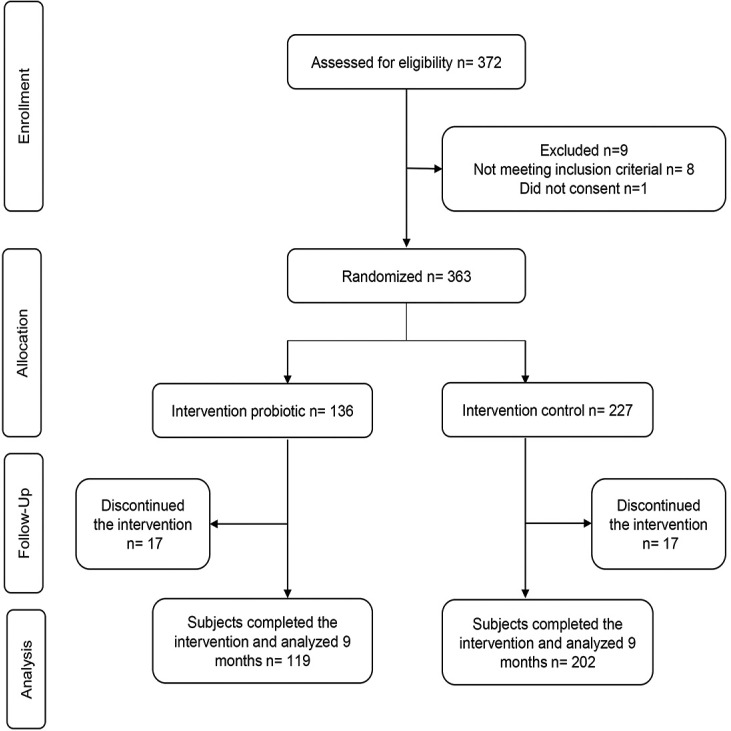
Flow chart of the randomization and attrition of participants throughout the 9-month study

The flow chart depicts the study population (Figure 1). Table 1 shows the baseline characteristics of the probiotic and control groups. There were no statistically significant differences between the groups, nor significant demographic differences between the children who continued in the study and those who left it.

**Table 1:** Baseline characteristics of children who participated in the study

Characteristic	Probiotic Group	Control Group	Dropouts
n	119	202	42
Female sex, %	50	47.6	20.1
Age (years), mean ± SD	3.48 ± 0.50	3.53 ± 0.42	3.80 ± 0.53
ICDAS 2-6>0, %	47.1	47.6	12.5
ICDAS 2 >0, %	38.2	40.5	45.2
ICDAS 3-4 >0, %	15.4	18.5	19.0
ICDAS 5-6>0, %	23.5	21.6	38.1
dICDAS2–6mft, mean ± SD	2.23 ± 3.57	2.22 ± 3.49	2.76 ± 3.81
dICDAS2mft, mean ± SD	1.07 ± 1.71	1.19 ± 1.92	1.57 ± 2.17
dICDAS3–4mft, mean ± SD	0.29 ± 0.79	0.71 ± 1.64	0.26 ± 0.63
dDCDAS5–6mft, mean ± SD	0.71 ± 1.64	0.65 ± 1.81	0.79 ± 1.47

### Effects on *S. mutans*, *Lactobacillus* spp., dental caries, dental plaque, pH, and salivary buffer capacity

The data of this study, as collected at baseline and at the end of the clinical trial, are shown in Table 2. The mean of the *S. mutans* CFU/mL was lower in the intervention group compared with the control group after 9 months, however, the differences did not reach statistically significance (*p*=0.173). The results showed statistically significant difference between intervention and control group in *Lactobacillus* spp. CFU/mL after intervention (*p*=0.002).

**Table 2:** *S. mutans*, *Lactobacillus* spp., dental caries in the probiotic group and control group at baseline and at 9 months of study

Variables	Probiotic group	Control group	p value	RR (95% CI)
Children development centers	5	5		
Participants	136	227		
**CFU/ml *S. mutans***				
Baseline (mean, sd)	0.84x10^5^ ± 3.13 x10^5^	1.02x10^5^ ± 3.89x10^5^	*p* = 0.200	
9 months (mean, sd)	0.16x10^5^ ± 0.80 x10^5^	0.42x10^5^ ± 2.86x10^5^	*p* = 0.173	
**CFU/ml *Lactobacillus* spp**				
Baseline (mean, sd)	6.53x10^5^ ±5.51x10^5^	5.87x10^5^ ±4.74x10^5^	*p* = 0.384	
9 months (mean, sd)	4.78x10^5^ ±4.15x10^5^	7.03x10^5^ ±7.00x10^5^	*p* = 0.002	
**Dental Caries lesions**				
ICDAS 2–6 >0, baseline, %	47.1	47.6	*p* = 0.924	
ICDAS 2–6 >0, 9 month, %	42.9	44.6	*p* = 0.767	
ICDAS 2, baseline, %	38.2	40.5	*p* = 0.666	RR= 1.039 [0.87-1.23]
ICDAS 2, 9 month, %	30.3	36.1	*p* = 0.282	RR= 1.092 [0.93-1.27]
ICDAS 5–6 >0, baseline, %	23.5	21.6	*p* = 0.667	
ICDAS 5–6 >0, 9 month, %	20.2	20.8	*p* = 0.894	
d(icdas2–6)mft, baseline, mean±SD	2.23 ± 3.57	2.22 ± 3.49	*p* = 0.910	
d(icdas2–6)mft, 9 month, mean±SD	2.22 ± 32.32	1.88 ± 30.57	*p* = 0.865	
d(icdas5–6)mft, baseline, mean±SD	0.71 ± 1.64	0.65 ± 1.81	*p* = 0.628	
d(icdas5–6)mft, 9 month, mean ± SD	0.60 ± 1.65	0.70 ± 1.80	*p* = 0.790	

ICDAS, International Caries Detection and Assessment System; SD, standard deviation. Boldface indicates statistical significance. p<0.05. RR; relative risk values (95% CI).

Dental caries decreased between baseline and at 9 months of intervention (dicdas2–6mft), in both the probiotic and the control group; however, these differences were not statistically significant (*p=*0.767). Regarding the probiotic group, the relative risk values (95% CI) for children with initial carious lesions were 1.03 (0.87-1.23, *p=*0.66) at the baseline and 1.09 (0.93-1.27, *p=*0.28) at 9 months. The ingestion of probiotics did not show a statistical significance in RR, indicating that there was no association between the consumption of probiotics and the appearance of initial caries during intervention (Table 2). The differences in dental plaque and pH in the probiotic and the control group were not significant after 9 months. There was statistically significant difference in the salivary buffering capacity at baseline and after 9 months of intervention between the probiotic and the control group (*p=0.00*) (Table 3).

**Table 3:** Dental plaque, pH, and salivary buffer capacity in the probiotic group and control group at baseline and at 9 months of study

Variables	Probiotic group	Control group	p value
Children development centers	5	5	
Participants	136	227	
**Plaque Index Median**			
MPI=0, baseline, %	61	67.4	p = 0.434
MPI=1, baseline, %	29.4	25.6	
MPI=2, baseline, %	9.6	7	
MPI=0, 9 month, %	81.5	70.8	p = 0.076
MPI=1, 9 month, %	15.1	21.3	
MPI=2, 9 month, %	3.4	7.9	
**pH**			
pH <7, baseline, %	76.5	75.3	p = 0.806
pH ≥7, baseline, %	23.5	24.7	
pH <7, 9 month, %	70.6	75.2	p = 0.361
pH ≥7, 9 month, %	29.4	24.8	
**Salivary buffering capacity**			
<3.5, baseline, %	36	57.7	p = 0.000
3.50 - 4.24, baseline, %	21.3	18.9	
4.24 - 4.75, baseline, %	5.2	7.9	
>4.75, baseline, %	28.7	15.4	
<3.5, 9 month, %	37.8	64.4	p = 0.000
3.50 - 4.24, 9 month, %	29.4	21.3	
4.24 - 4.75, 9 month, %	15.2	9.4	
>4.75, 9 month, %	17.6	5	

SD = standard deviation. Boldface indicates statistical significance. p < 0.05

## Discussion

This study determined whether the daily drinking of milk complemented with the probiotics *Lactobacillus rhamnosus* and *Bifidobacterium longum* decreased the CFU/mL of *S. mutans*, *Lactobacillus* spp., dental caries, and dental plaque and increased pH and salivary buffer capacity compared with standard milk in preschool children. The dropout rate was 11.6%. A total of 42 children abandoned the study; the participants withdrew because of the change of residence address and because they were compelled to enter primary school by Colombian law. Therefore, the age of the retired children is higher than the average of those who remained in the study. The dropout rates were 11% in the control group and 12.5% in the intervention group. This study did not record side effects associated with the bacteriotherapy with *Lactobacillus rhamnosus* and *Bifidobacterium longum.*

Cluster randomization was made for this trial. Since the number of children *per* group was different for each Child Development Center, the total number in the control group was greater than the intervention group. In accordance to the notebook filled by a supervisor with the help of the teachers, children from the probiotic group attended 89% and those from the control group attended 85% of trials, showing a high adherence to the treatment. During the last 2 weeks of December and the first of January the children did not receive the therapy because of the holyday season. The same is true of the Holly Week in April.

The two brands of milk used in this project, Nestlé Nan Pro 3® and Alpina Baby + Plus 3®, had similar nutritional characteristics in terms of total fat, total carbohydrates, sugars, proteins, and percentages of vitamins and minerals. The most relevant difference between the two brands was the presence of probiotic strains in Nestlé Nan Pro 3®. Therefore, the differences observed in the trial might be due to the probiotics instead of differences of milk brands. The probiotic strains in this study were based in *Lactobacillus rhamnosus* 5x10^6^ CFU/g in 62.5% and the *Bifidobacterium longum* 3x10^6^ CFU/g corresponding to 37.5%. *Lactobacillus rhamnosus* have shown antagonistic activity against *S. Mutans,* the primary cause in caries onset^17^ ^,^ ^19^. *Bifidobacterium longum* has been studied along with *Lactobacillus rhamnosus* ^11^ in the decrease of *S. Mutans.* We chose food supplemented with probiotics because it was the only one available in Colombia containing *Lactobacillus rhamnosus* in high percentage for consumption in preschool children.

### S. mutans

Studies such as the one performed by Näse, et al.^17^ (2001) found a decrease in counts of *S. Mutans* in preschoolers, when *Lactobacillus rhamnosus* is administered to the milk consumed at weekdays for 7 months; however, as in this study that provided milk for 9 months, the results are not statistically significant; the study of Stecksen-Blicks, Sjöström, and Twetman^22^ (2009) also in preschoolers provided *Lactobacillus rhamnosus* with 2.5 mg of fluoride *per* liter of milk for 21 months, and found coincident outcomes with the study of Näse, et al.^17^ (2001) and of this study: however, it is unknown if the effect of the decrease in the counts of *S. mutans* was due to the probiotic strain or the addition of fluoride in the milk.

The clinical trial of Tehrani, et al.^25^ (2016) found that the use of drop including *Lactobacillus rhamnosus, Bifidobacterium infantis,* and *Lactobacillus reuteri* for 2 weeks decreased significantly the *S. Mutans* salivary counts after an intervention in 3-6-year-old children. The studies of Tehrani, el al.^25^ (2016), Yadav, et al.^29^ (2014), Jindal, et al.^11^ (2011) and Campus, et al.^5^ (2013) are consistent in showing statistically significant reduction in the *S. Mutans* salivary counts between the intervention group and the control group after probiotic therapy; however, note that the intervention limited periods of these studies ranged from 1.4 weeks (10 days) to 6 weeks. Furthermore, the strains of probiotic bacteria used were different from those used in this study, except for one of the three groups of children assessed by Jindal, et al.^11^ (2011). The ages of the children studied were higher than those of this study and the vehicles of administration were different; therefore, extrapolating from these results to this study is not appropriate.

Various mechanisms of action were proposed for the workings of probiotics bacteria, but none have been universally accepted. Local and systemic effects are usually defined. Nevertheless, probiotics are not capable to continually establish in the oral cavity, thus, daily consumption is necessary to achieve its positive effects. This is an important aspect to consider when testing the potential effects of the probiotic^4^ ^,^ ^9^.

### *Lactobacillus* spp

The present intervention had an influence on the levels of *Lactobacillus* spp. in saliva; the prevalence decreased by the consumption of the *Lactobacillus rhamnosus* and *Bifidobacterium longum* containing milk. The study by Stecksen-Blicks, Sjöström and Twetman^22^ (2009) found that *Lactobacillus* spp. counts remained constant in preschool children with the consumption of *Lactobacillus rhamnosus* added with fluoride after 21 months of intervention; however, a confounding result from the fluoride summed to the probiotic group cannot be eliminated. Tehrani, et al.^25^ (2016) showed that the use of drop containing *Lactobacillus rhamnosus*, *Lactobacillus reuteri,* and *Bifidobacterium infantis* for 2 weeks had no effect on *Lactobacillus* spp. counts in preschool children. Cogulu, et al.^6^ (2010) observed a statistically significant reduction in salivary *S. mutans* and *Lactobacillus* spp. after 3 weeks of intervention with probiotic-kefir, a product of the fermentation of milk and cultures prepared from grains added with a multi strain probiotic in 104 subjects aged 20-27 years. Nonetheless, we must consider that this study was performed in young adults and the extrapolation must be made with caution. This clinical trial is a pioneer in showing these results for our knowledge, considering the specificities of the study, such as the age of the children, the probiotic strains, the duration, and the vehicle used to administer the probiotic. There are no reports of studies on the effects of *Lactobacillus rhamnosus* and *Bifidobacterium Longum* on the counts of *Lactobacillus* spp. in preschool children. The combination of these two probiotic bacteria, which are highly studied and tested for their benefits in the oral cavity^21^, may provide for superior therapeutic effectiveness than a single strain. Diversity in the oral environment allows a greater opportunity to overcome the barriers present by the host and its endogenous microorganisms^6^.

### Presence of dental caries lesions

The 9-month intervention time was short for full caries to progress. The probiotic group mainly and the control group to a lesser extent both decreased the prevalence of initial caries after nine months of probiotics therapy; however, this is not statistically significant. The results found in preschool children by Näse, et al.^17^ (2001) and Rodriguez, et al.^19^ (2016) show a significant decrease in caries lesions at the end of the intervention with *Lactobacillus rhamnosus* in milk at 7 and 10 months, respectively*;* Stecksén-Blicks, Sjöström and Twetman^22^ (2009) found the same results; however, these results added fluoride to milk, making it impossible to isolate the effect of the *Lactobacillus rhamnosus*; Hedayati-Hajikand, et al.^10^ (2015) found the same results in caries with 138 healthy 2-3-year-old children with an intervention of 12 months with Probiora 3® chewing tablets (*Streptococcus uberis* KJ2, *Streptococcus oralis* KJ3, *Streptococcus rattus* JH145).

The results of this study regarding dental caries can be explained in epidemiological studies in children and adolescents that have showed a lower incidence of dental caries to higher consumption of milk^1^ ^.^ Milk is a protein food that supplies indispensable amino acids and organic nitrogen for human beings. Milk also includes constituents that have protective attributes against caries, particularly calcium, phosphorus, and buffering capacity of the milk protein. The benefits of milk in caries control can be explained by several aspects, such as the remineralization of the enamel, the impediment of bacterial adhesion to the tooth and the obstruction in the formation of the bacterial biofilm^27^.

### Dental plaque

In this study, the average plaque index showed a better trend in children belonging to the group treated with probiotic therapy. Values of the zero plaque index were much higher in this group, although they did not reach statistical significance; Burton, et al.^3^ (2013), in a randomized, double-blind, placebo-controlled trial in 5-10 year old children, after 3 months of treatment with lozenges added to probiotic strain *Streptococcus salivarius* M18, found statistically significant decrease in plaque in comparison to that of the control group; similar results found Karuppaiah, et al.^12^ (2013), in 108 adolescents who consumed curds for a month. It is understood that, to the extent that probiotic strains reduce bacterial counts associated with dental caries, there is a decrease in plaque, which is where the cariogenic bacteria harbor.

### pH

Slight changes in the salivary acid reduction of preschool children were found after 9 months of milk supply with probiotics in this study; in the group that consumed standard milk, pH values were the same after 9 months of intervention. Campus, et al.^5^ (2013) found in 6-8-year-old children that the supply of *Lactobacillus brevis* CD lozenges after 6 weeks of intervention were effective in reducing plaque acidogenicity; these results were statistically significant at the end of the intergroup intervention. No studies were found in preschoolers that compared pH values after an intervention with probiotics.

### Salivary buffering capacity

The intervention in preschool children with the daily drinking of milk supplemented with *Lactobacillus rhamnosus* and *Bifidobacteruim longum* improved the buffer capacity of saliva in a consistent way, contributing to mitigate this risk factor for the appearance and progression of ECC^2^. No studies were found in preschoolers that compared the salivary buffering capacity after an intervention of milk with probiotics, nevertheless, the advantages of incorporating probiotics into dairy products because of the buffering capacity of the food matrix and the presence of milk components are found in the correlation with tooth enamel remineralization^30^.

For the authors, this research is the first randomized clinical trial that shows the effectiveness of *Lactobacillus rhamnosus* and *Bifidobacteruim longum* in the salivary buffer capacity in preschool children. The data are new and original.

The limitations of this clinical trial were the randomization of the groups of the Child Development Centers, which could pose a risk of bias, and the shortened period for follow-ups of intervention

## Conclusion

The daily consumption of milk supplemented with *Lactobacillus rhamnosus* and *Bifidobacteruim longum* reduces the CFU/mL *Lactobacillus* spp. counts and increases the buffer capacity of saliva in preschool children. Additional investigations are however required to explain the mechanisms of action of probiotics bacteria and to evaluate the cost-benefit of a public health measure with this evidence in mind.
